# Optimization-based Dielectric Metasurfaces for Angle-Selective Multifunctional Beam Deflection

**DOI:** 10.1038/s41598-017-12541-x

**Published:** 2017-09-25

**Authors:** Jierong Cheng, Sandeep Inampudi, Hossein Mosallaei

**Affiliations:** 1 Institute of Modern Optics, Nankai University, Key Laboratory of Optical Information Science and Technology, Ministry of Education, Tianjin, 300071 China; 2 Department of Electrical and Computer Engineering, Northeastern University, 360 Huntington Ave, Boston, Massachusetts 02115 USA

## Abstract

Synthesization of multiple functionalities over a flat metasurface platform offers a promising approach to achieving integrated photonic devices with minimized footprint. Metasurfaces capable of diverse wavefront shaping according to wavelengths and polarizations have been demonstrated. Here we propose a class of angle-selective metasurfaces, over which beams are reflected following different and independent phase gradients in the light of the beam direction. Such powerful feature is achieved by leveraging the local phase modulation and the non-local lattice diffraction via inverse scattered field and geometry optimization in a monolayer dielectric grating, whereas most of the previous designs utilize the local phase modulation only and operate optimally for a specific angle. Beam combiner/splitter and independent multibeam deflections with up to 4 incident angles are numerically demonstrated respectively at the wavelength of 700 nm. The deflection efficiency is around 45% due to the material loss and the compromise of multi-angle responses. Flexibility of the approach is further validated by additional designs of angle-switchable metagratings as splitter/reflector and transparent/opaque mirror. The proposed designs hold great potential for increasing information density of compact optical components from the degree of freedom of angle.

## Introduction

The behavior of electromagnetic waves through an interface has long been governed by the constitutive parameters of natural materials until the appearance of metasurfaces^1–3^. Serving as a class of artificial boundaries, metasurfaces produce desirable electromagnetic responses over an optically thin layer of space-variant resonators and scatterers. Such breakthrough concept provides a smart way of complex wavefront shaping and has advanced rapid development of flat devices^4^ with superior and diverse functionalities, such as lenses^5,6^, wave plates^7–9^, holograms^10–12^, and cloaks^13–16^ with great potential for revolutionizing conventional optical components.

Driven by the continuous demand of system integration and device minimization, incorporating more and more functionalities in a single metasurface platform is highly demanding. For practical applications in communication, energy harvesting, imaging and sensing areas, control over light according to its fundamental properties (wavelengths, polarizations and directions) is usually needed. Multifunctional metasurfaces aiming at dual/multi frequency bands or with polarization-selective features have been extensively studied^17–21^. They rely on spatial multiplexing of different subarrays, either in-plane^17–19^ or layered^20,21^, in a shared aperture with each subarray imparting a specific phase map. The inherent properties of light (wavelengths or polarizations) are the “keys” to unlock different functionalities without coupling or interfering among information channels. Functions for the orthogonal polarization states are naturally independent^22–28^. For multiwavelength metasurfaces, separation of the wavelength windows needs to be large enough to prevent coupling among different wavelengths^17,18,29^. Multiple information channels are superimposed into a single platform by changing shapes, dimensions and orientations of the meta-atoms and meta-molecules^30^.

Angle-sensitive functionality, another critical feature demanded in many optical systems such as solar cells and light emitters/detectors, but unfortunately, has not been extensively explored yet in the metasurface platform^31^. Existing methods for angular filtering based on geometrical optics^32^ and Brewster angles^33,34^ are bulky and still far away from subwavelength-scale manipulation. Control of light by angle over an extremely thin layer of metasurface seems quite challenging. The difficulty may exist in two aspects. First, most of the metasurface inclusions are not sensitive to angle due to their subwavelength thickness, especially for plasmonic nanoantennas^35,36^. In addition, beams with different incident angles are strongly coupled. One cannot separately design subarrays for different angles and merge them together in segment or interleaved fashion, as one functionality may cast severe background noise to another.

On the other hand, the well-known generalized Snell’s law opens up an unique way for anomalous beam deflection by introducing phase discontinuities along an interface^37^. Although the phase shifters are designed to provide a desired phase gradient for a specific incident direction (e.g. normal excitation), they show the same phase gradient for a wide range of incident angles (around normal direction)^37,38^. This phenomenon is favorable for wide-view applications, but on the other hand limits the freedom of angular selectivity, and forces waves from different directions to follow the same deflection rule.

**Figure 1:**
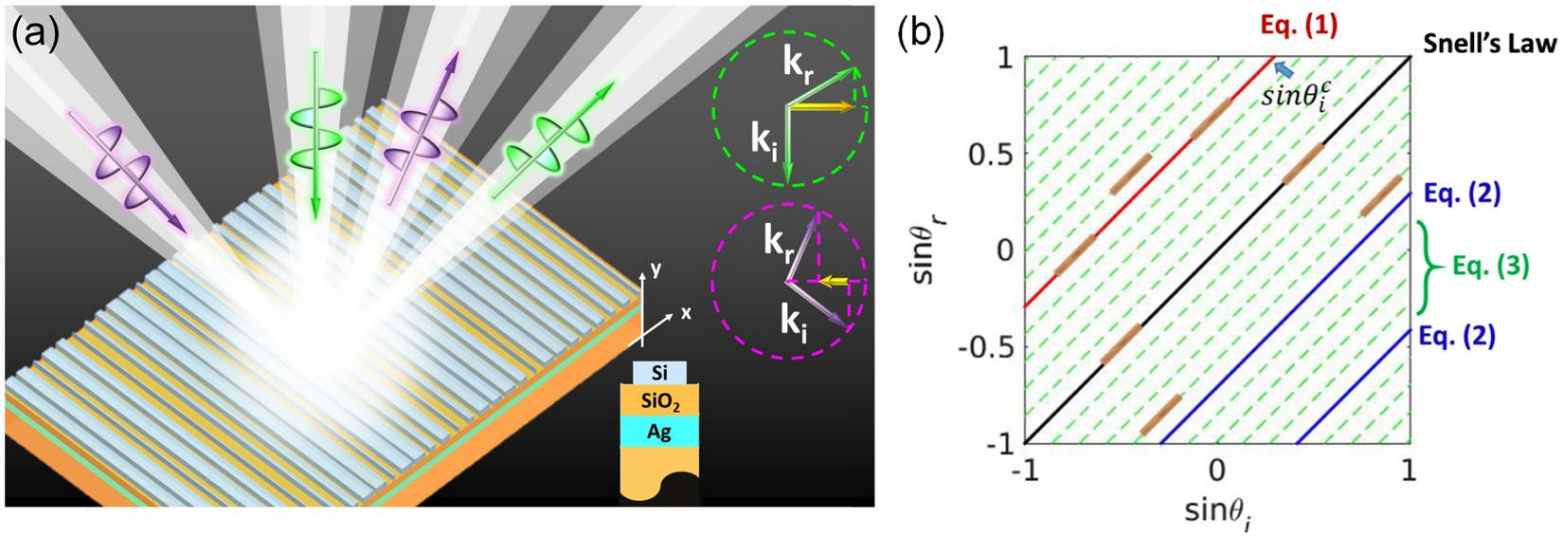
(**a**) Schematic metagrating for angle-selective beam deflection. Different phase gradients can be seen by beams from different directions, leading to independent beam deflections controlled by angle. The momentum conservation is illustrated in the upper right corner, with the metasurface phase gradient represented by the yellow arrow. The reflective grating element is shown in the lower right corner. (**b**) Snell’s law of reflection and its generalization. (*ξ* = *k*_0_/$\sqrt{2}$ and *m* = 5 are considered)

Here we propose a dielectric monolayer metagrating in Fig. 1(a) with angle-controlled arbitrary deflection functions. Without adding any complexity to the geometry, such metagrating imparts multiple independent phase gradients according to the incident angle (as indicated by the upper right inset of Fig. 1(a)) due to the well-designed interaction of light with phase gradient and lattice periodicity, with great potential to increase the information capacity of the metadevices from a new degree of freedom: direction. To bypass the single phase gradient functionality imparted by the local phase approximation, we choose an alternative metasurface design methodology based on inverse numerical optimization techniques. Although this study is focused on one-dimensional reflection engineering, the angle-selective concept is general and extensible to two-dimensional deflection in either reflection or transmission side by optimizing two-dimensional gratings.

## Results

It is well-known that the reflection angle is equal to the incident angle over a homogeneous interface based on Snell’s law. The relation of the angles is simply expressed as the diagonal black line in Fig. 1(b). When a constant phase gradient *ξ* is introduced by the metasurface at the interface, beams coming into and out of the metasurface are governed by the generalized law of reflection^37^, also known as the grating equation^39^:

1 $${k}_{0}(sin{\theta }_{r}-sin{\theta }_{i})=\xi $$

where *k*_0_ is the wavevector in vacuum, and *θ*_*i*_ and *θ*_*r*_ are angles of incidence and reflection. The angles are defined with respect to the surface normal, with positive values if the tangential wavevector is along positive direction and vice versa. The artificially introduced phase gradient brings a vertical shift of the relation between *sinθ*_*i*_ and *sinθ*_*r*_ as the red line in Fig. 1(b). Such shift is accompanied by a critical incident angle ${\theta }_{i}^{c}=asin(sgn(\xi )-\xi /{k}_{0})$ beyond which the reflected beam is expected to couple into evanescent wave^37,40^. As observed from several experiments^37,38^, beams coming from different directions within the critical range do follow the red line in Fig. 1(b) with the same phase gradient. However, beyond the critical angle, different behaviors are observed in plasmonic and low-loss acoustic metasurfaces^40,41^. The light stays in the evanescent mode in plasmonic metasurfaces due to the intrinsic loss^40^, whereas large portion of the energy is re-directed into propagation directions in acoustic metasurfaces^41^, as a result of the negligible system loss and the strong non-local interaction with the supercell lattice. The supercell periodicity of the metasurface comes from the folding of the monotonically increasing or decreasing phase profile, which adds an amended term to Eq. (1) when the metasurface operates beyond ${\theta }_{i}^{c}$ ^41–43^:

2 $${k}_{0}(sin{\theta }_{r}-sin{\theta }_{i})=\xi +nG=\pm \frac{2\pi }{p}\pm n\frac{2\pi }{p}$$

where *p* is the supercell periodicity covering 2*π* phase profile, and *G* is the reciprocal lattice vector. *ξ* and *G* have the same value of ±2*π*/*p*, but different physical meanings, with the former as the phase gradient and the latter as the effective lattice wavevector^42^. Thus *n* is an integer representing the diffraction orders. Within the critical angle, *n* = 0, light is bended due to the linear phase jump; beyond the critical angle, *n* may take values of −1, −2 $\cdots$ (1, 2 $\cdots$) if *ξ* is positive (negative). In the later case, the lattice wavevector will compensate the phase gradient, and redirect the beam into propagation directions. These possible directions fall into the black line as conventional specular reflection and blue lines as apparent negative reflection in Fig. 1(b). All the previous metasurfaces are optimized only for beam steering within the critical regime. The response beyond the critical angle is unpredictable and uncontrollable: the beam may be diffracted into one or multiple diffraction orders. In the case of *θ*_*i*_ beyond ${\theta }_{i}^{c}$, the metasurface is not efficient for wavefront shaping, and the reflection direction cannot be arbitrarily designed as the supercell is totally fixed based on the need of reflection within the critical regime.

In this work, we further extend the generalized Snell’s law by designating different values to *ξ* and *G*:

3 $${k}_{0}(sin{\theta }_{r}-sin{\theta }_{i})=\xi +nG=\pm \frac{2\pi }{p}\pm n\frac{2\pi }{mp}$$

where one lattice period contains *m* supercells of the same size *p* with different geometries but the same phase gradient with respect to a certain impinging direction. The phase gradient *ξ* stays the same as in Eq. (2) along the surface, and the lattice wavevector *G* is only 1/*m* of *ξ*. For clarity, *p* is the length of the phase supercell (PSC), and *mp* is the length of the lattice supercell (LSC). The more PSCs are included in one LSC, the more diffraction orders *n* may interact with the incoming light. The large LSC (small G) fills the gaps between *n* and *n* ± 1 diffraction orders with additional (*m* − 1) states, which are shown as the green dash lines in Fig. 1(b). Here each LSC contains 5 different PSCs as an example. It offers large flexibility to control the reflection as a function of angles by leveraging the effects of phase gradient and lattice periodicity. One may optimize the LSC configuration to selectively enhance different diffraction orders according to the incident angles, just as the discrete brown lines indicated in Fig. 1(b).

Eq. (3) can also be considered as the multi-order grating^44^ equation working with high diffraction orders instead of the traditional first diffraction order. However, our focus here is to achieve angle-selective diffraction orders. The separation of the local refraction and the lattice diffraction in Eq. (3) helps defining multiple PSCs in one LSC. As illustrated next, the freedom in choosing PSCs is the key to enable angle-selective beam deflection.

### Manually designed metasurfaces for dual-angle reflection

To further understand the concept in Eq. (3), we manually design two types of metasurfaces with different LSCs, both bending the normal incident beam (*θ*_*i*_ = 0°) into *θ*_*r*_ = 45°. LSC is the same as PSC in the first design, while LSC involves 2 PSCs in the second structure. The basic metasurface element consists of a silicon bar on top of the silver back mirror with a SiO _2_ slab sandwiched in between. The mirror has a fixed thickness of 100 nm to block any light at the working wavelength of *λ*_0_ = 700 nm throughout this work. The spacer layer is 110 nm thick with the permittivity of 2.25. The silicon bar has a fixed height of 220 nm with the permittivity following Green and Keevers^45^. As mentioned above, the dielectric bars benefit from low material loss at visible frequency band and better sensitivity to angle than plasmonic bars. Such choice of inclusions is a key step towards success of angle-selective functions. The metegrating sits in the x-z plane with inhomogeneous pattern along x direction, schematically shown in Fig. 1(a). TM polarization is considered in this work with the magnetic field parallel to the bar direction (H_*z*_).

**Figure 2:**
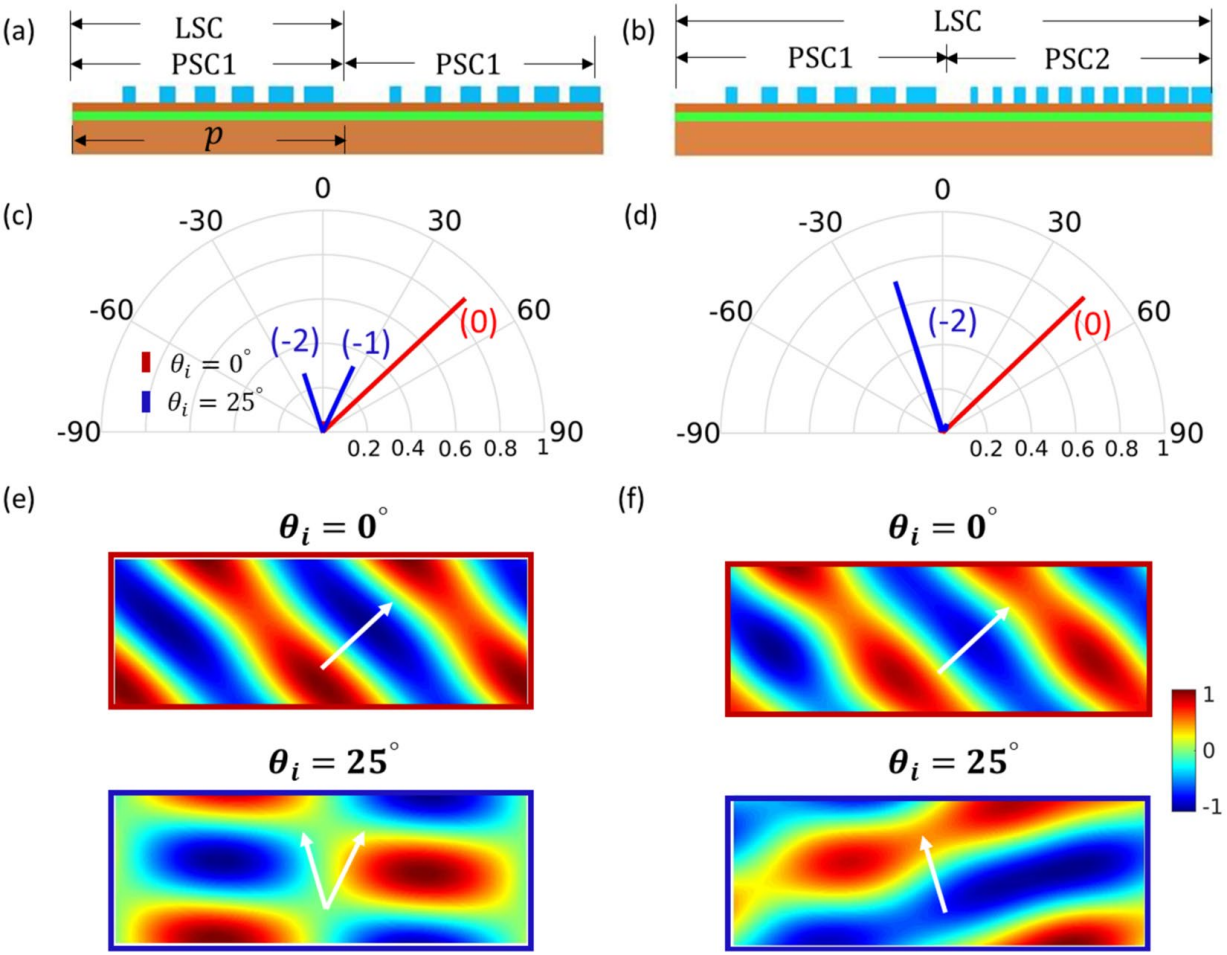
Comparison of metasurfaces when the LSC includes one PSC in (**a**) and two PSCs in (**b**). The PSC1 is achieved by locally changing the bar width with the fixed bar-to-bar distance of 140 nm. In PCS2, the bar-to-bar distance is 80 nm. The thickness of the bar and the spacer layer are fixed as 220 nm and 110 nm. (**c**) and (**d**): far field patterns when the beams with *θ*_*i*_ = 0° (red line) and *θ*_*i*_ = 25° (blue line) are reflected by the two metasurfaces. (**e**) and (**f**): reflected near field (H_*z*_) from the metagratings (**a**) and (**b**). The grating sits at the bottom in each plot. Arrows are for eye guidance.

Steering the beam from 0° to 45° requires the phase gradient *ξ* = *k*_0_/$\sqrt{2}$ along the metasurface plane. A linear phase profile with the aforementioned gradient is achieved in a PSC by monotonically increasing the width of the bar while fixing the bar-to-bar distance as 140 nm. We mark it as PSC1. Another PSC can be readily designed with the same phase profile by fixing the bar-to-bar distance as a different value, 80 nm in this example, which is marked as PSC2. One metasurface is composed of PSC1 only in a periodic fashion, and the other repeats every 2 PSCs: PSC1 and PSC2, as shown in Fig. 2(a) and (b).

Rigorous coupled wave analysis (RCWA)^46^ is used to characterize the two metasurfaces. As observed from the far field responses in Fig. 2(c) and (d), both bend the normal incident beam into 45° with 90% efficiency, as the phase functions are exactly the same for normal incident beam in the two designs. Nevertheless, two structures behave differently when the beam comes from another angle beyond the critical value (${\theta }_{i}^{c}{\mathrm{=17}}^{\circ }$). When *θ*_*i*_ = 25°, the first metasurface splits the beam into two directions 25° and −16.5° corresponding to *n* = −1 and *n* = −2 in Eq. (2), each carrying 30% of the input energy. The second metasurface redirects 70% of the energy into −16.5°. The near fields (reflected field H _*z*_) for *θ*_*i*_ = 0° and *θ*_*i*_ = 25° are plotted for the two metasurfaces in Fig. 2(e) and (f), with a good agreement with the far field responses. In RCWA, the field is the summation of plane waves with different spatial harmonics according to the lattice periodicity. The directions in the near fields are determined by checking the amplitude and the direction of each harmonic plane wave. By incorporating two PSCs into one LSC, one gain additional control over the responses for additional angle. As more PSCs are properly taken into the LSC, multifunctionality is achievable over more incident angles, and simultaneously, with increased flexibility in choosing the deflection angles due to the denser diffraction order distribution. In such case, systematic inverse geometry optimization is a better choice than manual design to fulfill balanced responses over angle.

### Independent multibeam deflection controlled by angle via geometry optimization

Next, we systematically develop a methodology to achieve independent and arbitrary beam deflection for multiple incident angles using optimized metagrating design. The operation wavelength, wave polarization and the grating materials are the same as in Fig. 2 and keep invariant throughout this work. We solve the inverse problem by first defining the deflection goals, and then find the metagrating geometry through joint analysis of RCWA and optimization algorithm.

Starting with two beams, one beam reflection is aimed from *θ*_*i*_ = 0° to *θ*_*r*_ = 45° and the other from *θ*_*i*_ = 30° to *θ*_*r*_ = 45°. Two beams are merged into the same direction. Such metasurface can be used as an ultrathin beam combiner/multiplexer. Please note that the two impinging beams are not restricted to inside and outside the critical angle. The input and output directions can be defined arbitrarily as long as they do not violate the reciprocity principle. The phase gradient for beam bending from *θ*_*i*_ = 0° to *θ*_*r*_ = 45° is *ξ* = *k*_0_/$\sqrt{2}$. The length of one PSC covering 2*π* phase delay for normal excitation is *p* = 2*π*/*ξ* = 990 nm. We define the LSC with 10 different PSCs, leading to the lattice periodicity of 10*p* = 9.9 *μ*m (≈14*λ*_0_) and the lattice wavevector of *G* = 2*π*/(10*p*). This lattice period is large enough (the lattice wavevector is small enough accordingly) to scatter light into almost any arbitrary direction with a diffraction angle step of 4°. In particular, the metasurface needs to follow *n* = 0 in Eq. (3) for 0° to 45° bending, and *n* = −7 for 30° to 45° bending with *m* = 10.

To achieve such dual-angle functionalities, we associate RCWA with the interior point optimization algorithm^47^ in MATLAB to design the metasurface LSC. Briefly, in RCWA, the fields in each layer of the metasurface are represented by the summation of spatial harmonics corresponding to the diffraction orders(bloch modes) defined by the periodicity of the grating. For homogeneous layers, the spatial harmonics are plane electromagnetic waves satisfying the dispersion relations that also represent the angle of propagation directly. Inside patterned layers the spatial harmonics are numerically computed by solving the eigenvalues of the wave equation. Mathematically, the fields in *p* ^*th*^ layer of the system are expressed as,

4 $$[\begin{array}{c}{E}_{x;p}\\ {H}_{z;p}\end{array}]=[\begin{array}{cc}{{\rm{\Phi }}}_{x} & 0\\ 0 & {{\rm{\Phi }}}_{x}\end{array}][\begin{array}{cc}{W}_{p} & -{W}_{p}\\ {V}_{p} & {V}_{p}\end{array}][\begin{array}{cc}{{\rm{\Phi }}}_{y;p}^{+} & 0\\ 0 & {{\rm{\Phi }}}_{y;p}^{-}\end{array}][\begin{array}{c}{i}_{p}\\ {r}_{p}\end{array}],$$

where, Φ_*x*_ is a diagonal matrix whose elements are phase factors along the tangential direction defined as ${{\rm{\Phi }}}_{x}^{(n,n)}=\exp (i{k}_{x}^{(n)}x)$ with ${k}_{x}^{(n)}={k}_{x0}+n2\pi /{\rm{\Lambda }}$. *k*_*x*0_ represents the central order. The quantity ${{\rm{\Phi }}}_{y;p}^{\pm }$ are also diagonal matrices that represent the phase factor along the propagation direction in the *p* ^*th*^ region, whose elements are defined as ${{\rm{\Phi }}}_{y;p}^{+(n,n)}=\exp (+i{k}_{y;p}^{(n)}(y-{y}_{p-1}))$ and ${{\rm{\Phi }}}_{y;p}^{-(n,n)}=\exp (-i{k}_{y;p}^{(n)}(y-{y}_{p}))$. If the *p* ^*th*^ region is a homogeneous layer, ${k}_{y;p}^{(n)}=\sqrt{{k}_{0}^{2}{\varepsilon }_{p}-{({k}_{x}^{(n)})}^{2}}$. If the *p* ^*th*^ region is an inhomogeneous layer, then ${k}_{y;p}^{(n)}$ are the square root of the eigenvalues of the matrix *A*, defined as,

5 $$A={k}_{0}^{2} {\mathcal E} -{K}_{x}{ {\mathcal E} }^{-1}{K}_{x} {\mathcal E} ,$$

where, ${K}_{x}$ is a diagonal matrix with ${K}_{x}^{(n,n)}={k}_{x}^{(n)}$ and ${\mathcal E}$ is a toeplitz matrix of fourier coefficients of spatial permitivity ${\varepsilon }_{p}(x)$ of the inhomogeneous layer, defined as ${ {\mathcal E} }^{(m,n)}={\varepsilon }_{m-n}$. $[{\varepsilon }_{n}={\int }_{-{\rm{\Lambda }}\mathrm{/2}}^{{\rm{\Lambda }}\mathrm{/2}}{\varepsilon }_{p}(x)\exp (in2\pi /{\rm{\Lambda }})dx]$. Similarly, if the ${p}^{th}$ layer is homogeneous, the quantities ${W}_{p}$ and ${V}_{p}$ are diagonal matrices with ${W}_{p}^{(n,n)}=-{k}_{y;p}^{(n)}/{k}_{0}{\varepsilon }_{p}$ and ${V}_{p}^{(n,n)}\mathrm{=1}$, else ${W}_{p}$ represents a matrix whose columns are the eigenvectors of the matrix *A* and ${V}_{p}$ is a matrix defined as ${V}_{p}={k}_{0} {\mathcal E} {W}_{p}{K}_{y;p}^{-1}$. The quantities ${i}_{p}$ and ${r}_{p}$ are column vectors representing the amplitude coefficients of the eigenmodes that are determined by the boundary conditions at the ${p}^{th}$ interface; ${H}_{z;p+1}(x,{y}_{p})={H}_{z;p}(x,{y}_{p})$ and ${E}_{x;p+1}(x,{y}_{p})={E}_{x;p}(x,{y}_{p})$. The net reflection matrix of the system relating ${i}_{1}$ and ${r}_{1}$ as ${r}_{1}=R{i}_{1}$ can be computed by solving the matrix equation iteratively using the procedure defined in ref.^48^. In more details, the matrix equation can be represented as

6 $$[\begin{array}{c}{r}_{-M}\\ \vdots \\ {r}_{-1}\\ {r}_{0}\\ {r}_{1}\\ \vdots \\ {r}_{n}\\ \vdots \\ {r}_{M}\end{array}]=[\begin{array}{ccccccccc}{R}_{-M,-M} & \cdots  & {R}_{-M,-1} & {R}_{-M\mathrm{,0}} & {R}_{-M\mathrm{,1}} & \cdots  & {R}_{-M,n} & \cdots  & {R}_{-M,M}\\ \vdots  & \vdots  & \vdots  & \vdots  & \vdots  & \vdots  & \vdots  & \vdots  & \vdots \\ {R}_{-\mathrm{1,}-M} & \cdots  & {R}_{-\mathrm{1,}-1} & {R}_{-\mathrm{1,0}} & {R}_{-\mathrm{1,1}} & \cdots  & {R}_{-\mathrm{1,}n} & \cdots  & {R}_{-\mathrm{1,}M}\\ {R}_{\mathrm{0,}-M} & \cdots  & {R}_{\mathrm{0,}-1} & {R}_{\mathrm{0,0}} & {R}_{\mathrm{0,1}} & \cdots  & {R}_{\mathrm{0,}n} & \cdots  & {R}_{\mathrm{0,}M}\\ {R}_{\mathrm{1,}-M} & \cdots  & {R}_{\mathrm{1,}-1} & {R}_{\mathrm{1,0}} & {R}_{\mathrm{1,1}} & \cdots  & {R}_{\mathrm{1,}n} & \cdots  & {R}_{\mathrm{1,}M}\\ \vdots  & \vdots  & \vdots  & \vdots  & \vdots  & \vdots  & \vdots  & \vdots  & \vdots \\ {R}_{n,-M} & \cdots  & {R}_{n,-1} & {R}_{n\mathrm{,0}} & {R}_{n\mathrm{,1}} & \cdots  & {R}_{n,n} & \cdots  & {R}_{n,M}\\ \vdots  & \vdots  & \vdots  & \vdots  & \vdots  & \vdots  & \vdots  & \vdots  & \vdots \\ {R}_{M,-M} & \cdots  & {R}_{M,-1} & {R}_{M\mathrm{,0}} & {R}_{M\mathrm{,1}} & \cdots  & {R}_{M,n} & \cdots  & {R}_{M,M}\end{array}][\begin{array}{c}{i}_{-M}\\ \vdots \\ {i}_{-1}\\ {i}_{0}\\ {i}_{1}\\ \vdots \\ {i}_{n}\\ \vdots \\ {i}_{M}\end{array}]$$

where each element of the matrix *R* represents the coupling coefficient between incident and reflected harmonics, or in other words, incident and reflected plane waves from different angles decided by the periodicity of the LSC. From Eq. (6), the rows in *R* correspond to the output harmonics with different wavevectors (angles), and the columns correspond to the input harmonics, with the total number of (2*M* + 1) harmonics considered. Any beam deflection from *θ*_*i*_ to *θ*_*r*_ is achievable by optimizing the geometry to maximize a specific reflection element **R**_*i*,*j*_ in the *j* ^*th*^ column where the *i* ^*th*^ row and the *j* ^*th*^ column represent the reflected diffraction angle and the incident diffraction angle. As can be seen, RCWA is a straightforward method for beam deflection engineering using inverse scattering methods as the propagation directions are directly related to the harmonics. And it is also an efficient method for multi-angle deflection optimization as **R** calculated once in each optimization step automatically takes into account multiple incident and reflected angles.

Back to the dual-angle beam combiner, we set *M* = 500 in the optimization so that the harmonic waves can resolve the grating width as small as *λ*_0_/20. The first beam deflection from 0° to 45° happens when ${{\bf{R}}}_{\mathrm{10,0}}$ is maximized in the 0^th^ column. And the second beam deflection from 30° to 45° corresponds to maximizing ${{\bf{R}}}_{\mathrm{10,7}}$ in the 7^*th*^ column. We define the objective function as Eq. (7).

7 $$objective=min(\sum _{k=-M}^{M}|{R}_{k\mathrm{,0}}-{\delta }_{k\mathrm{,10}}{|}^{2}+\sum _{k=-M}^{M}|{R}_{k\mathrm{,7}}-{\delta }_{k\mathrm{,10}}{|}^{2})$$

where *δ* is the Kronecker delta function. The nonlinear multi-variable function ‘fmincon’ in MATLAB is utilized to minimize the objective function based on the reflective Newton methods. The free parameters of the optimization are totally 68 geometry parameters in one LSC: width of bars and air gaps (33 pairs), the height of bars (same for all the bars), and the height of the spacer layer, while the thickness of the back mirror is fixed as 100 nm. The total width of all the bars and gaps is constrained to 9.9 *μ*m, length of one LSC. The lower boundary for the width of bars and air gaps is set to be 10 nm considering the fabrication constraints. The LSC with 10 repeated PSCs are used as the initial guess. The function ‘fmincon’ searches for a local minimum within the defined bounds. Hence the net dimensions of the geometries are always in the limit of fabrication. The process is carried with a limit of 6 million iterations for each free parameter to target a tolerance level of 10^−1^. However, the local minimum is always found before reaching the maximum number of iterations.

**Figure 3:**
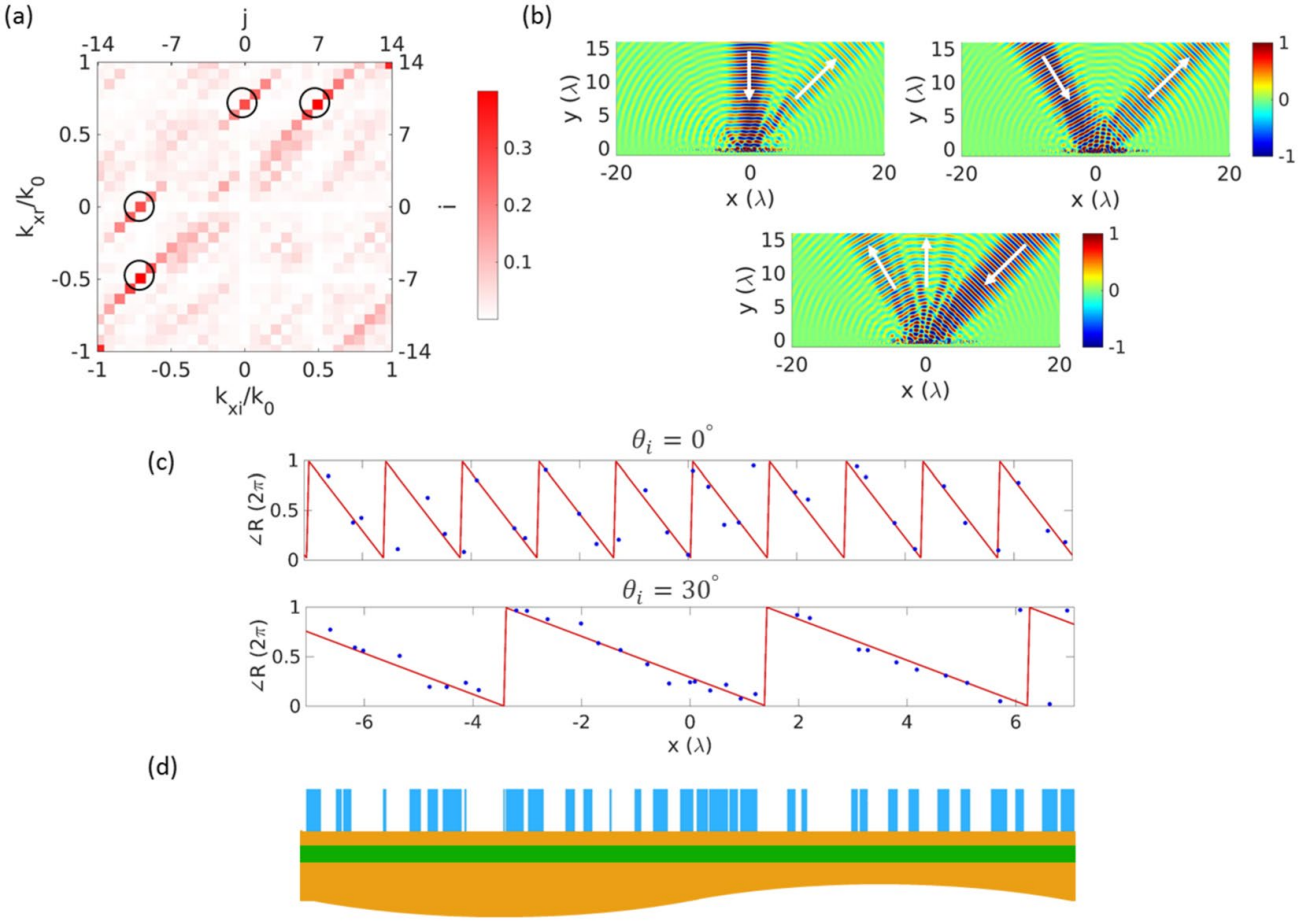
(**a**) Reflection matrix $|{\bf{R}}{|}^{2}$ for beam combiner and beam splitter following the principle of reciprocity. (**b**) Incident and reflected magnetic field H_*z*_. Both normal and 30° beams are reflected into the same direction 45°. The same grating splits a single beam from ${\theta }_{i}=-{45}^{\circ }$ into two beams along directions of ${\theta }_{r}\,=\,{0}^{\circ }$ and ${\theta }_{r}=-{30}^{\circ }$ with the power ratio of 40:60. (**c**) Reflection phase profile when the incident beam is from 0° and 30°. Lines are the desired values, and dots are achieved by the grating elements. (**d**) Geometry of the optimized grating LSC with bar thickness of 284 nm and spacer thickness of 80 nm.

The optimized reflection matrix |**R**|^2^ with the meaning of diffraction efficiency is shown in Fig. 3(a) as functions of input and output tangential wavevectors within the propagating regime. The diffraction efficiencies go to maximum at the desired positions marked by the upper two circles, which are 30% for ${\theta }_{i}\,=\,{0}^{\circ }$ and 40% for ${\theta }_{i}\,=\,{30}^{\circ }$. One can clearly see two minimized vertical lines (white color) going through the maximized elements, indicating extremely low noises along undesired directions. The signal-to-noise ratio (SNR) is calculated to be 20 dB for this dual-angle beam deflection. The matrix in Fig. 3(a) is a specific paradigm of the angle-dependent beam steering concept illustrated in Fig. 1(b).

Near field response of the optimized metagrating is studied in Fig. 3(b). A finite metagrating is used by repeating the LSC 3 times with Gaussian beam excitation. The Gaussian beam has a narrow beam waist of 3*λ*_0_ in order to separate the incident and reflected beams for clarity. It is evident that the normal and 30° incident beams are both reflected into 45° direction. High directivity is further validated here for both beam steerings. Following the optical reciprocity principle, this metagrating splits the −45° incident beam into two beams along 0° and −30° as expected in Fig. 3(b). This beam-splitting feature is also indicated in Fig. 3(a) by the lower two circles due to the symmetry of the matrix.

The optimized metasurface LSC obtaind from the inverse scattering procedure is shown in Fig. 3(d) with the bar thickness of 284 nm and spacer thickness of 80 nm. Detailed geometrical dimensions of the grating are included in Supplementary Information for verification and fabrication consideration. There are few bars and gaps which are extremely narrow, with the width approaching the optimization lower boundary 10 nm. Our simulation shows that the grating still functions without the extremely narrow bars and slits. To make the geometry more practical, one can further optimize the grating pattern with the width limit corresponding to the fabrication resolution. On top of the grating is the reflection phase shift profile offered by those bars. The top panel of Fig. 3(c) is for normal excitation, and the bottom panel is for oblique 30° excitation. The solid lines are the ideal phase maps needed for steering the two beams. The dots are the phase shift given by each grating bar, which are generally in good agreement with the ideal values. Different phase gradients are expressed in the same metagrating for two beam angles. If one considers that each 2*π* phase variation covers one PSC in top panel of Fig. 3(c), the LSC involves 10 different PSCs as expected.

The optimized grating shows the efficiency of 70% as beam splitter (summation of the two lower circles in Fig. 3(a)). The loss comes from two aspects: inherent material loss in silicon bars and undesired scattering due to angle compromise. Silicon is dispersive and lossy at the working wavelength of 700 nm. If the material loss is ignored, our design shows that the efficiency can be improved to 82%, very close to the theoretical limit of the optimal beam splitting approach^49^. In addition, we observe from Fig. 3(c) that not all the bars offer the desired phases for both beams. Some elements work better for one incident beam and sacrifice for the other beam. Such compromise between the two incident angles is another factor limiting the deflection efficiency. The relatively low efficiency for individual beam bending from 0° and 30° is due to the reciprocity constraint, where each beam steering has a maximum efficiency limit of 50%, with the left power going into the evanescent diffraction orders. As we will see in the next section, the efficiency for each beam bending is improved if they are bent to different directions.

Although there exist some works on grating beam splitters^50,51^, metasurface designs as beam splitters working in one polarization have not been extensively studied. Here the angle between the separated two beams is designable by choosing target diffraction orders. In addition, the splitting ratio is adjustable by giving different weights to each summation term in Eq. (7). The beam splitter in Fig. 3 has a splitting ratio around 40:60.

### Angular capacity and versatile angle-selective functionalities

**Figure 4:**
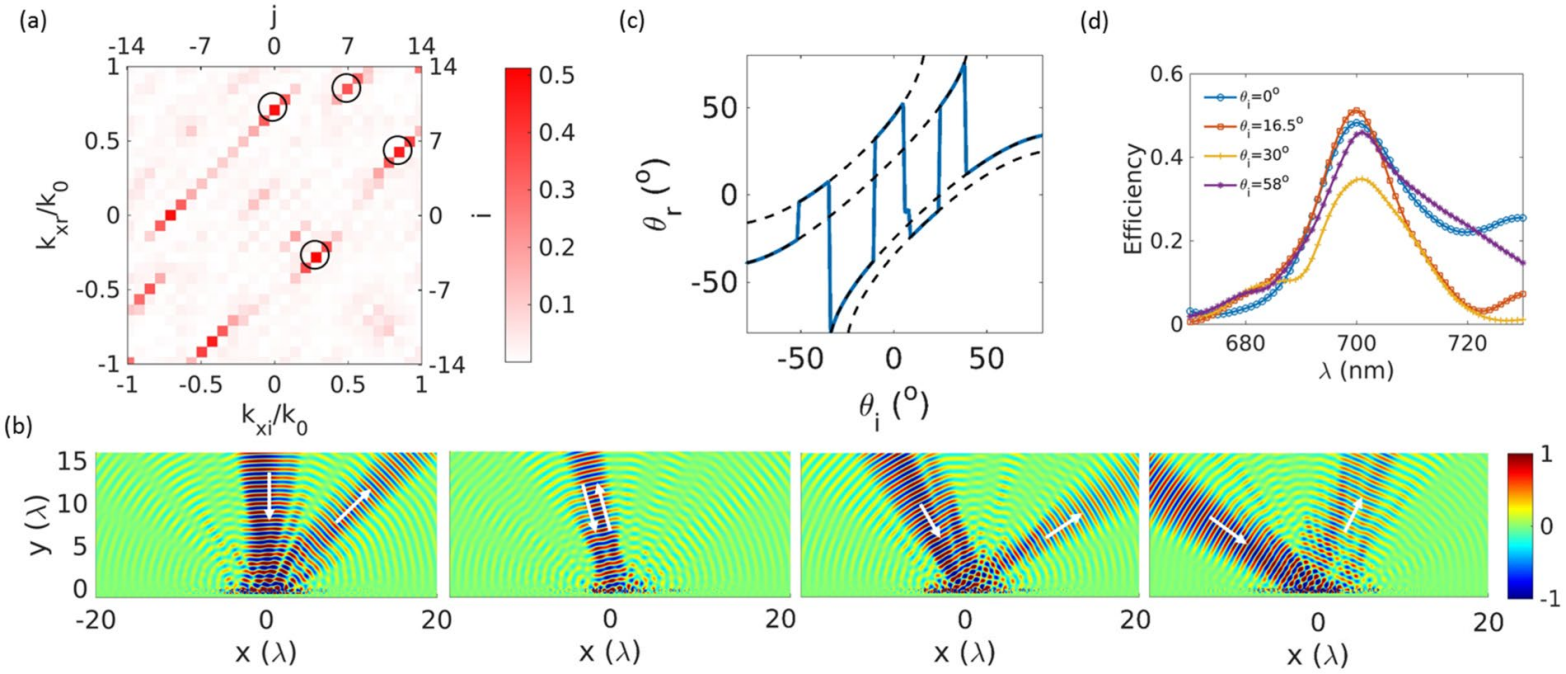
Four-angle beam deflection in a single metagrating. (**a**) The optimized reflection matrix $|{\bf{R}}{|}^{2}$ with the target elements marked by circles. (**b**) Near fields. The beams from ${\theta }_{i}\,=\,{0}^{\circ }{\mathrm{,16.5}}^{\circ }{\mathrm{,30}}^{\circ }{\mathrm{,58}}^{\circ }$ are reflected into ${\theta }_{r}\,=\,{45}^{\circ },\,-{16.5}^{\circ },\,{58}^{\circ },\,{25}^{\circ }$, following four different phase gradients. The grating sits in the y = 0 plane. (**c**) Variation of the deflection angle of the main beam as the incident angle sweeps from −90° to 90° (solid line). Dash lines are the anomalous reflection following Eq. (3) when $n\,=\,\mathrm{0,}\,-\mathrm{18,}\,-\mathrm{5,}\,-16$. (**d**) Variation of the deflection efficiency at different wavelengths for four beams.

To explore the capability of a single metagrating for multibeam deflection with increased number of angle channels, optimization is done for four impinging beams with incident angles of 0°, 16.5°, 30° and 58° using the same LSC size of 9.9 *μ*m. The grating is optimized to deflect the four aforementioned beams into different target diffraction orders, $n\,=\,\mathrm{0,}\,-\mathrm{18,}\,-\mathrm{5,}\,-16$ in Eq. (3) with $p\,=\,3.3$ *μ*m and *m* = 10. The optimization turns out to be maximizing four elements in the reflection matrix as marked by the circles in Fig. 4(a). The near field responses are detailed in Fig. 4(b). All the beams are reflected into the desired directions with the average deflection efficiency of 45% (62% averaged efficiency if the material loss is ignored in the bar layer). The dimensions of this metagrating can be found in Supplementary Information.

As the incident wave sweeps from −90° to 90° in Fig. 4(c), the deflection angle of the main beam is segmented following the desired four phase gradients. The transition of the main beam direction is quite sharp. With previously designed metasurfaces, beam deflection always follows one dash line in Fig. 4(c), and being out of control after the cutoff of such line (critical angle). Here the deflection can jump freely among different gradients, leading to rich angle-selective responses. In addition, the plot in Fig. 4(c) shows only the direction of the main beam. The total deflected field does not change directions in such a sharp manner. The far field deflection patterns for incident beams from 0° to 16.5° with 5° steps are shown in Fig. S1 of the Supplementary Information. The deflection gradually moves from one diffraction order to another as the incident angle varies. Between two optimized incident windows (0° and 16.5°), the deflection may happen in both diffraction orders and even other undesired diffraction orders.

Metasurface designed by such optimization method deflects beam into different diffraction orders according to incident directions. Selection of diffraction orders should not violate the reciprocity principle, as validated by the symmetric reflection matrix along diagonal in Figs 3(a) and 4(a). In addition, strong crosstalk may exist if the input beam directions are densely distributed. The low sidelobe level (SNR $\,\approx \,18$ dB) in Fig. 4(a) indicates that the angle separation of 15° is large enough to block channel mixing. Design with denser angle channels might be challenging, as each segmentation in Fig. 4(c) governs the deflection within at least 15° (3 diffraction orders) around the target direction. From physical point of view, the grating inclusions do not have enough freedom in the optimization process to offer distinct phase responses for closely orientated beams. Larger LSC size with more PSCs included helps to further increase the number of angle channels. Multi-layer design should also be a solution for angle-dense functionalities.

As the wavelength moves away from the designed 700 nm, the deflection efficiency of the four beams decreases (Fig. 4(d)), indicating the narrow bandwidth. However, due to the low material dispersion of the dielectric grating, the proposed design can be easily scaled to other wavelengths.

**Figure 5:**
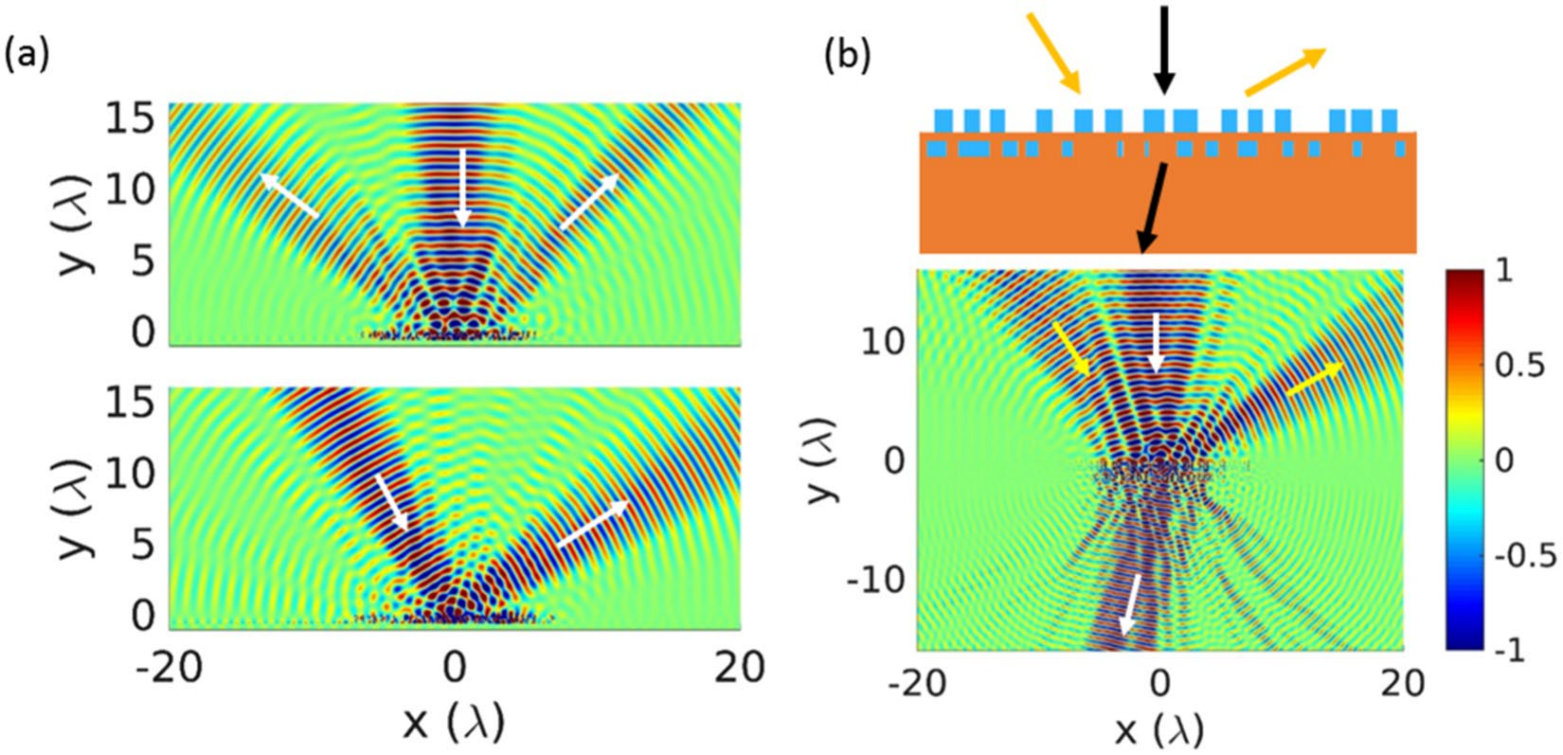
(**a**) Metagrating as beam splitter and reflective mirror with the function switchable by the incident angle. (**b**) Bi-layer metagrating optimized for selective transmission and reflection. The lattice supercell is shown on top of the field plot. The top and bottom bar layers are separated by 200 nm spacer with the bar thickness of 600 nm and 400 nm, respectively.

To further verify the flexible and versatile capability of the proposed method in angle-selective wavefront engineering, additional designs are incorporated in Fig. 5. Figure 5(a) shows a single layer grating with two switchable performances as beam splitter and reflective mirror according to the incident angle. Coming from normal direction, the beam splits into two beams along −50° and 45° with splitting ratio of 50:50, whereas the beam is totally reflected if the excitation comes from 30°. This dual-functionality is achieved by selectively maximizing two elements in the normal incident column and one element in the 30° incident column in the reflection matrix, with the deflection efficiency around 45%. More importantly, the optimization method is not limited to the reflection mode. The same angle-selective multifunctionality can be achieved in the transmission mode by optimizing the transmission matrix similarly. One can even engineer the reflection and transmission matrices simultaneously to control reflection and transmission according to angles. In Fig. 5(b), by removing the back mirror and using bi-layer grating design, with one layer embedded in the substrate and the other layer in air, we gain even larger flexibility to engineer the wavefront in both reflection and transmission modes. The normal excitation directly goes through with the transmission angle steerable (${\theta }_{t}=-{20}^{\circ }$ in this case), and the oblique excitation is reflected from ${\theta }_{i}\,=\,\,{30}^{\circ }$ to ${\theta }_{r}\,=\,{58}^{\circ }$. The efficiencies for the transmitted and reflected beams are 53% and 74%, respectively. This is a thin film with transparency controlled by angle, a desirable feature in many display applications.

## Discussion

Finally, we claim that although most of the previous metasurfaces consist of only one set of PSCs, one may come up with numerous types of PSCs with the same phase gradient for a specific incident angle by properly choosing the bar width *w* and the bar-to-bar distance Λ. For example, varying Λ among different PSCs and varying *w* within each PSC; varying *w* among different PSCs and Λ within each PSC; or varying both in each PSC.

**Figure 6:**
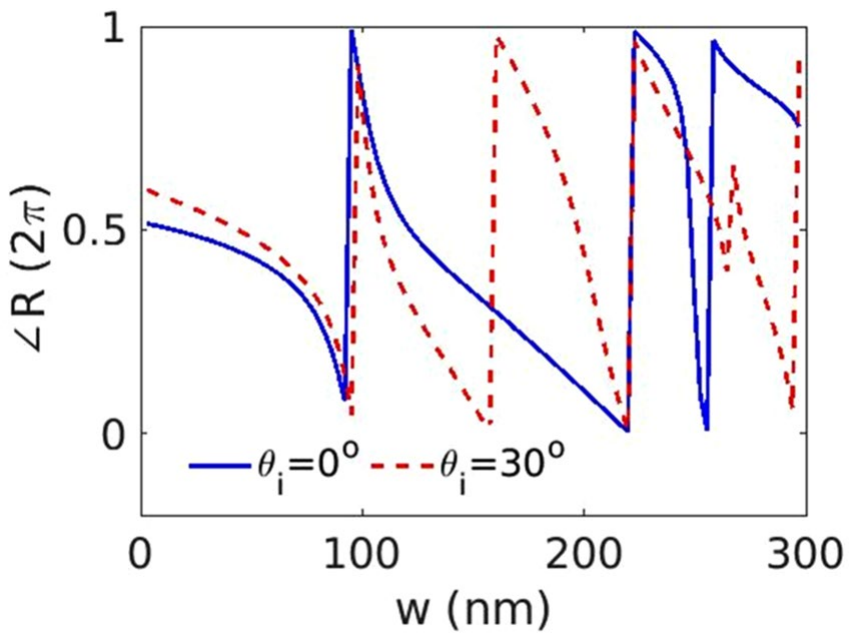
Reflection phase of periodic bars as a function of the bar width *w* with normal and 30° excitation. The bar-to-bar distance is fixed as ${\rm{\Lambda }}\,=\,300$ nm. The thickness of the Si bar and the SiO_2_ slab are 200 nm and 80 nm, respectively.

In addition, as indicated in Fig. 6, multiple 2*π* phase shifts are covered by sweeping the bar width *w*, which offers additional degree of freedom in designing different PSCs. This is a unique feature of dielectric resonators compared to the plasmonic ones, but at the expense of relatively large thickness. From Fig. 6, one has several choices of *w* which give the same phase response for normal excitation, but different phase responses for 30° oblique excitation. These different choices of the phase resonators lay the foundation to achieve angle-dependent performances, but without adding any complexity into the grating geometry and without significant efficiency degradation for multibeam deflection up to 4 incident angles. A recent work about dispersion engineering over a continuous bandwidth shares similar principle^52^. Beams either with continuous wavelengths or with different angles are strongly coupled states, and the multifunctionality is available by systematically optimizing all the geometric parameters.

## Conclusion

In this work, we proposed an innovative methodology with extreme flexibility to generate angle-dependent phase gradients over a monolayer dielectric metagrating for efficient multibeam deflection, by incorporating several phase supercells into one lattice supercell. An inverse scattering method based on combination of RCWA and numerical optimization offers an efficient way to design the grating texture by linking the reflection matrix to the target beam direction straightforwardly. The local phase variation and the non-local lattice scattering together selectively enhance the wave in a desired diffraction order according to different incident angles. Two-beam combiner/splitter and independent deflection with up to four angle channels are successfully demonstrated with high efficiency. Switchable features of splitter/reflector and transparent/opaque film are also explored according to different beam directions. The concept here can be easily extended to two-dimensional beam deflection using grating blocks, working in either reflection or transmission mode. Just as the anomalous beam deflection has flourished numerous complicated wavefront engineering over metasurfaces, we expect such angle-selective deflection will be generalized for more complicated wavefront engineering, such as angle-dependent focusing and hologram, leading to advances in multifunctional chip-scale devices in a variety of application fields.

## Electronic supplementary material

Geometry Details
