# Supplementary material for: Optimization-based Dielectric Metasurfaces for Angle-Selective Multifunctional Beam Deflection
Source: Sci Rep. 2017 Sep 25;7:12228. doi: 10.1038/s41598-017-12541-x (PMC5613029; doi:10.1038/s41598-017-12541-x)
Supplement: Supplementary file 1 — Geometry Details [file 41598_2017_12541_MOESM1_ESM.pdf]

Supplementary Information:

Optimization-based Dielectric Metasurfaces for Angle-Selective Multifunctional Beam Deflection

Jierong Cheng, Sandeep Inampudi, and Hossien Mosallaei*

Department of Electrical and Computer Engineering, Northeastern University, Boston, Massachusetts, 02115, United States

*E-mail: hosseinm@ece.neu.edu

1. The metagrating in Fig. 4 of the main manuscript deflects beams from 4 directions into different diffraction orders. Here the far field is plotted for the first two beams with incident angles of 0° (Fig. S1(a)) and 16.5° (Fig. S1(d)). The beams coming from directions between the two optimized ones are also plotted in Fig. S1(b) and (c) to clarify how the diffraction order is changed.
   First, the two beams under optimization with incident angles of 0° and 16.5° have the deflection efficiency of around 50%, with 22% and 15% of power distributed in all the undesired diffraction orders in Fig. S1 (a) and (d), respectively, and the left absorbed by the material loss (both silicon and mirror).

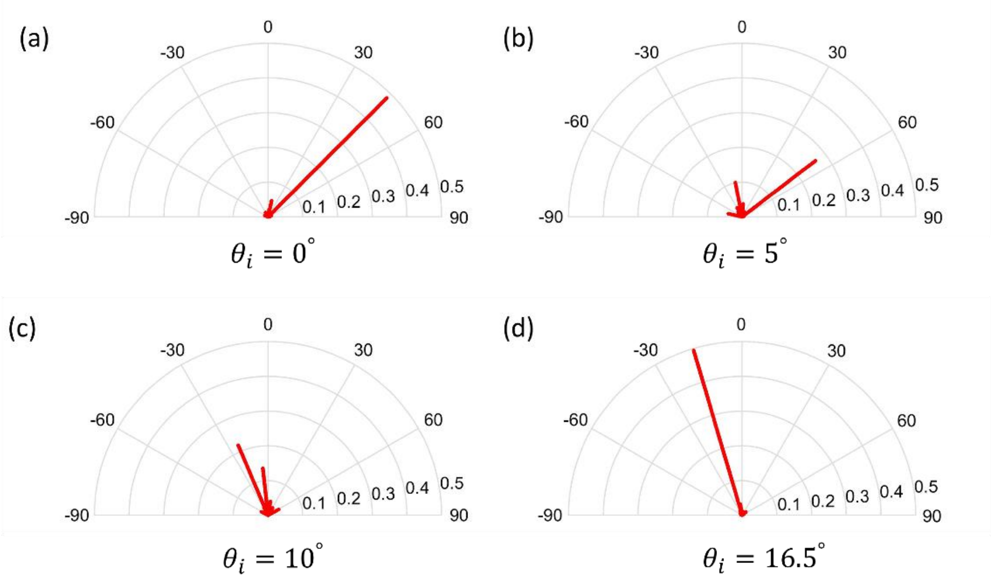

Fig. S1 Far field patterns of the beams with different incident angles reflected by the four-beam metagrating in Fig. 4 of the main manuscript. The incident directions are 0°, 5°, 10°, 16.5° in (a) to (d).

   In addition, one can observe that moving from one diffraction order to another is not a jump, but a gradual process. The main beam in Fig. S1(b) follows the diffraction order of Fig. S1(a), and the main beam in Fig. S1(c) follows the diffraction order of Fig. S1(d). The sidelobe in Fig. S1(b) increases and becomes the main beam in Fig. S1(c).

2. Width of bars and air gaps of the metagrating lattice supercell in Figure. 3 of the main text with the bar thickness of 284 nm, spacer layer thickness of 80 nm and back mirror thickness of 100 nm.

	width (nm)
gap	215
bar	193
gap	182
bar	79

gap	13
bar	110
gap	391
bar	46
gap	289
bar	146
gap	81
bar	135
gap	57
bar	243
gap	30
bar	29
gap	459
bar	24
gap	10
bar	231
gap	50
bar	200
gap	271
bar	120
gap	103
bar	120
gap	212
bar	30
gap	285
bar	89
gap	140
bar	192
gap	150
bar	171
gap	40
bar	146
gap	10
bar	244
gap	10
bar	114
gap	23
bar	221
gap	372
bar	111
gap	70

bar	77
gap	548
bar	89
gap	19
bar	104
gap	256
bar	122
gap	134
bar	135
gap	230
bar	154
gap	135
bar	126
gap	258
bar	209
gap	97
bar	113
gap	222
bar	203
gap	35
bar	175

3. Width of bars and air gaps of the metagrating lattice supercell in Figure. 4 of the main text with the bar thickness of 300 nm, spacer layer thickness of 90 nm and back mirror thickness of 100 nm.

	width (nm)
gap	196
bar	187
gap	187
bar	215
gap	197
bar	114
gap	22
bar	163
gap	148
bar	232
gap	169
bar	123

gap	271
bar	54
gap	18
bar	280
gap	110
bar	53
gap	251
bar	196
gap	99
bar	178
gap	128
bar	182
gap	133
bar	76
gap	130
bar	122
gap	211
bar	41
gap	311
bar	130
gap	182
bar	149
gap	42
bar	168
gap	129
bar	224
gap	127
bar	85
gap	203
bar	182
gap	254
bar	124
gap	75
bar	100
gap	246
bar	103
gap	102
bar	59
gap	262
bar	74
gap	235

bar	104
gap	302
bar	92
gap	85
bar	113
gap	202
bar	120
gap	257
bar	168
gap	10
bar	195
gap	98
bar	103
